# Inhibition of miR-191 contributes to radiation-resistance of two lung cancer cell lines by altering autophagy activity

**DOI:** 10.1186/s12935-015-0165-5

**Published:** 2015-02-04

**Authors:** Zhenkuan Liu, Shaoxiang Huang

**Affiliations:** Department of Respiratory, Tianjin Fifth Central Hospital, 41 Zhejiang Road, Tianjin, China

**Keywords:** Lung cancer, Autophagy, miR-191, Radiation-resistant

## Abstract

**Background:**

Lung cancer is the leading cause of cancer-related morbidity and mortality all over the world. Surgery resection, radiotherapy, chemotherapy, immunotherapy and combined treatments have been discovered and well established for treatments. However, low survival rate of five years after clinical treatments mainly due to recurrence of stress-resistant cancer cells calls for better understanding and new ideas. Our project aimed to understand the forming process of stress resistant lung cancer cells after radiotherapy.

**Methods:**

Two classic non-small cell lung cancer (NSCLC) cell lines A549 and H1299 initially were radiated with a ^137^Cs gamma-ray source with doses ranging from 0 to 12 Gy to generate radiation-resistant cancer cells. 8 Gy of radiation was regard as a standard dosage since it provides effective killing as well as good amount of survivals. The expression levels of autophagy-related proteins including Beclin-1, LC3-II and p62 were studied and measured by both western blot and quantitative real-time polymerase chain reaction (real-time RT-PCR).

**Results:**

Increased Beclin-1, LC3-II and decreased p62 have been observed in radiation-resistant cells indicating elevated autophagy level. Decreased miR-191 in radiation-resistant cells performed by Taqman qRT-PCR also has been seen. Two binding sites between Beclin-1 and miR-191 suggest potential association between.

**Conclusions:**

It is reasonable to speculate that inhibition of miR-191 expression in lung cancer cells would contribute to the establishment of radiation-resistant cells via mediating cellular autophagy. Therefore, miR-191 is a potential target for therapy in treating radiation-resistant lung cancer.

## Introduction

Lung cancer is the leading cause of cancer-related mortality and morbidity and it is one of the predominant life-threatening conditions among cancers. For 50 years both the morbidity and mortality rates from many countries have increased significantly [1]. However, the exact cause underlying lung cancer is still unrevealed. Improvements in long term survival rate have been achieved by early diagnosis and combinations of chemo/radiotherapy and surgery. However, the recurrence rate is still high and five years survival for NSCLC patients remains as low as 15% [2]. One main factor could be the resistant cancer cells after certain therapies.

For now, there is no medicine shown to be significantly effective and consistent to treat lung cancer. When feasible, surgical resection is still the single most effective and successful option especially for early-stage patients [3-5]. There are two main types of lung cancer, small cell lung cancer (SCLC) and non-small cell lung cancer (NSCLC). NSCLC accounts for around 84% of all the cases. NSCLC patients are more likely to be cured with surgery resection if discovered at an early stage [6]. Radiotherapy, chemotherapy, immunotherapy and combined treatments would be chosen based on tumour stage and histologic type based on accurate diagnosis. Radiotherapy is the most important localised treatment across every clinical phase of NSCLC patients. However, the records of the past two decades shown that only 5-10% of patients survived 5 years after clinical therapy [7]. Malissard et al. has shown that there is a tight correlation between recurrence and distant metastasis [8], therefore recurrence is the main cause responsible for failed treatment and the occurrence of metastasis of NSCLC. The recurrence is frequently associated with stress-resistant cancer cells after therapies [9]. A study focusing on 598 stage I NSCLC patients who underwent surgery resection illustrated around 27% of overall recurrence incidences [10]. Our project aimed to understand the forming process of stress-resistant lung cancer cells after radiotherapy. In our experiment, two classic NSCLC cell lines A549 and H1299 were chosen. Survived cells with three week’s radiation were used as the cell model after radiotherapy for further experiments.

Autophagy is a catabolic process to breakdown and recycles dysfunctional cellular components such as organelles and proteins by isolating these selected components inside a double membrane-bound vesicle. This function is used as a survival mechanism by starving cells [11,12]. When it comes to cancer cells, it could maintain the metabolism of mitochondrial, improve cell proliferation and increase stress tolerance. All of these effects together would eventually contribute to the level of malignance. Additionally, it may interfere with chemotherapy and radiation-induced apoptosis through removal of damaged organelles [13]. These evidences suggest the role autophagy might play in tumor development [14]. It hints that inhibition of autophagy activity is the effective way to suppress the development of cancer. MicroRNA is a single-stranded small sequence of non-coding RNA molecule with length of 20–24 nucleotides which accounts for post-transcriptional regulation of gene expression [15,16]. MicroRNA could silence gene expression by specific binding through base pairing with complementary mRNA sequence followed by degradation of the target mRNA [17]. Therefore it plays a crucial role in regulation of gene expression, cell cycling and developmental timing [18]. It is estimated that miRNAs modulates over one-third of human genes and various biological pathways [19]. Some studies illustrated degradation of miRNA might contribute to the developments of various diseases including cancer [20]. The link established between autophagy and miRNA is the possible regulation function of miRNA upon autophagy [20]. Dysfunctional miRNAs are frequently found in malignancies where they function as either oncogenes or tumor suppressors [21]. MiRNA has been suggested to prevent the development of lung cancer by inhibiting some specific function proteins involved in autophagy pathway. So far some researches have pointed out the potential correlation between dysfunctions of miRNA-186, 143 [22], 17–92 cluster [23,24] and the progress of lung cancer. In this report, we also found the reduced expression level of miRNA-191 in two types of NSCLC based on miRNA microarray techniques. However, the link between miRNA-191 and autophagy has not been reported in other studies. Taken together, we propose that the inhibition mechanism to lung cancer by miRNA-191 is conducted by targeting specific proteins of autophagy pathway, which eventually inhibit the growth and proliferation of lung cancer cells. The phenomenon could be helpful for therapy of lung cancer in the future.

## Results and discussion

Various studies have illustrated the significant role autophagy plays during the progress of tumor cells, including representing cell survival mechanism as well as tumor suppression. In our experiments, autophagy related proteins such as Beclin-1 and LC3-II were found to be upregulated in two NSCLC radiation-resistant cell lines, indicating enhanced level of autophagy in radiation-resistant cancer. Increasing amount of studies were performed to understand tumor biology from microRNA profile perspective.

### Establishment of radiation-resistant cancer cells

**Figure 1:**
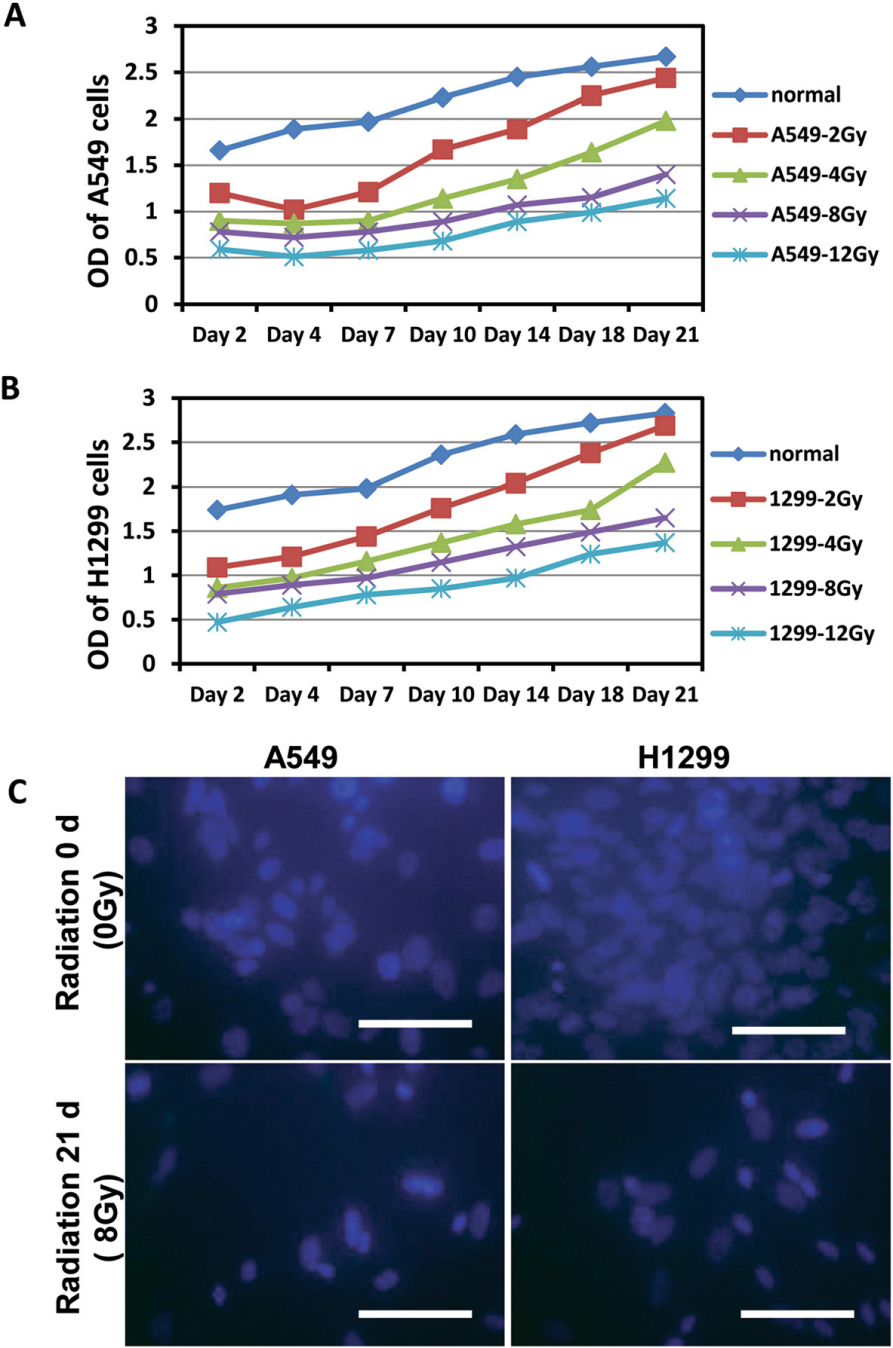
**The radiation survived cells have the growth capacity for a long time. (A)** OD values of A549 cells. **(B)** OD values of H1299 cells. **(C)** Normal A549 and H1299 cells and the cells treatment with 8 Gy radiation after 3 weeks were stained by DAPI (blue) and images were acquired under fluorescence microscopy.

Two non-small cell lung cancer cell lines A549 and H1299 were chosen for this project. In order to investigate the role microRNA might play in the forming process of radiation-resistant cancer cells during radiotherapy, normal A549 and H1299 cells were treated with ^137^Cs gamma-ray source with dosages ranging from 0, 2, 4, 8 to 12 Gy. ODs measured (Figure 1A and B) on different days show an increasing trend, which illustrated gradual growth of surviving cells after radiation. The results showed the killing effect was radiation dosage-dependent on both cell lines. 8 Gy radiation dosage was selected as a standard since then because it kills fairly enough cells and also provides good amount of survived cells for further experiments. Figure 1C shows the images generated from fluorescent dye DAPI staining and it clearly demonstrate much more living cells survived after radiation treatment comparing with the cells on 21 d. Taken together, we can conclude that radiation treatment effectively kills both A549 and H1299 cells. As expected, there was a small fraction of cells radiation, which closely mimiced the clinical radiotherapy-resistant lung cancer cases. This population of radiation-resistant cells might eventually lead to the recurrence of cancer. The radiation-resistant models established by using the treatment discussed above would be utilized for further experiments.

### Autophagy-associated protein profile

**Figure 2:**
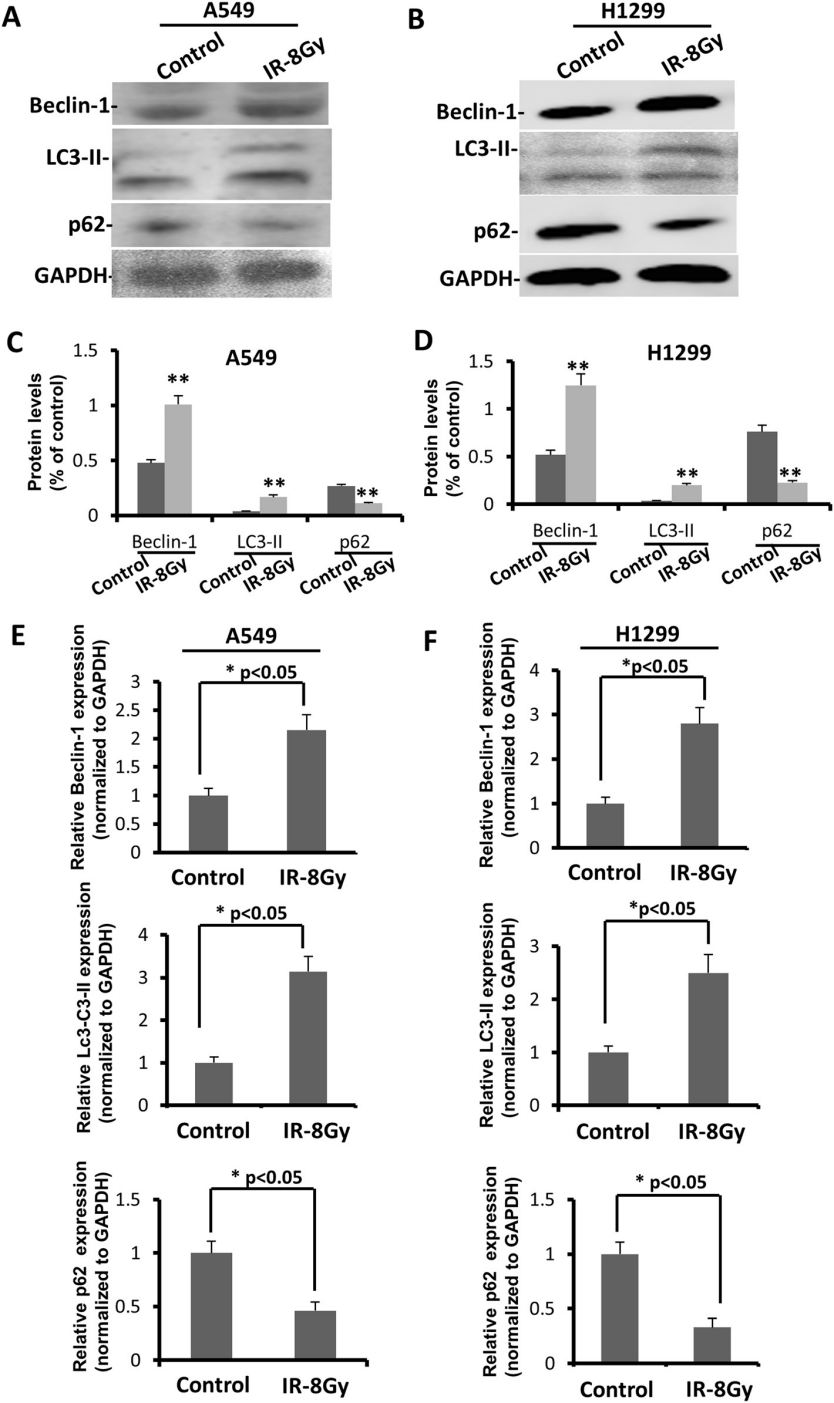
**Autophagy upregulated in radiation resistant A549 and H1299 compared in non-radiation resistant A549 and H1299. (A, C)** Expression status of certain autophagic marker proteins Beclin-1, LC3-II, p62 and GAPDH (loading control) in non-treatment A549 and radiation treatment A549 cells. **(B, D)** Expression status of certain autophagic marker proteins Beclin-1, LC3-II, p62 and GAPDH (loading control) in non-treatment H1299 and radiation treatment H1299 cells. **(E)** Relative Beclin-1, LC3-II and p62 mRNA expression levels in normal A549 and radiation resistant A549 cells, bars represent mean ± S.E. (*p <0.05 vs control, n = 3). **(F)** Relative Beclin-1, LC3-II and p62 mRNA expression levels in normal H1299 and radiation resistant H1299 cells, bars represent mean ± S.E. (*p <0.05 vs control, n = 3).

The second part of this project is to study expression profiles of autophagy associated proteins in radiation-resistant cells. Beclin-1 is an autophagy marker protein in human [25,26] which is also involved in cell death, neurodegeneration, tumorigenesis and development [27]. Overexpressed Beclin-1 has been observed in gastric cancer cells from previous study [28]. A type of Beclin-1 mutant resulted in reduced autophagy and progressed tumor growth in a NSCLC tumor xenograft model [29]. LC3-II is another autophagic marker that is usually down-regulated in human lung cancers [30]. p62 is a signaling adaptor, which accounts for Ras-induced survival and transformation and is also found to be overexpressed in tumor cells [31]. The increased expression levels of autophagy associated Beclin-1 and LC3-II and reduced p62 level have been observed in radiation-resistant group in comparison to control group in both lung cell lines (Figure 2A-D). In order to analyze precisely the differences of expression levels between radiation resistant and untreated cancer cells, relative mRNA levels of Beclin-1, LC3-II and p62 were also measured by qRT-PCR. PCR results shown in Figure 2C and D also confirmed elevated level of both Beclin-1 and LC3-II and decreased p62. These changes of autophagy associated protein expression levels indicated enhanced level of autophagy in radiation resistant cancer cells. One study demonstrated that knockdown of *Beclin-1* and another autophagy related gene *ATG5* lead to higher sensitivity to radiation, suggesting cytoprotective function of induced autophagy [32]. This might also explain why enhanced level of autophagy has been observed in radiation-resistant cancer cells during our experiment.

### MicroRNA profiles

**Figure 3:**
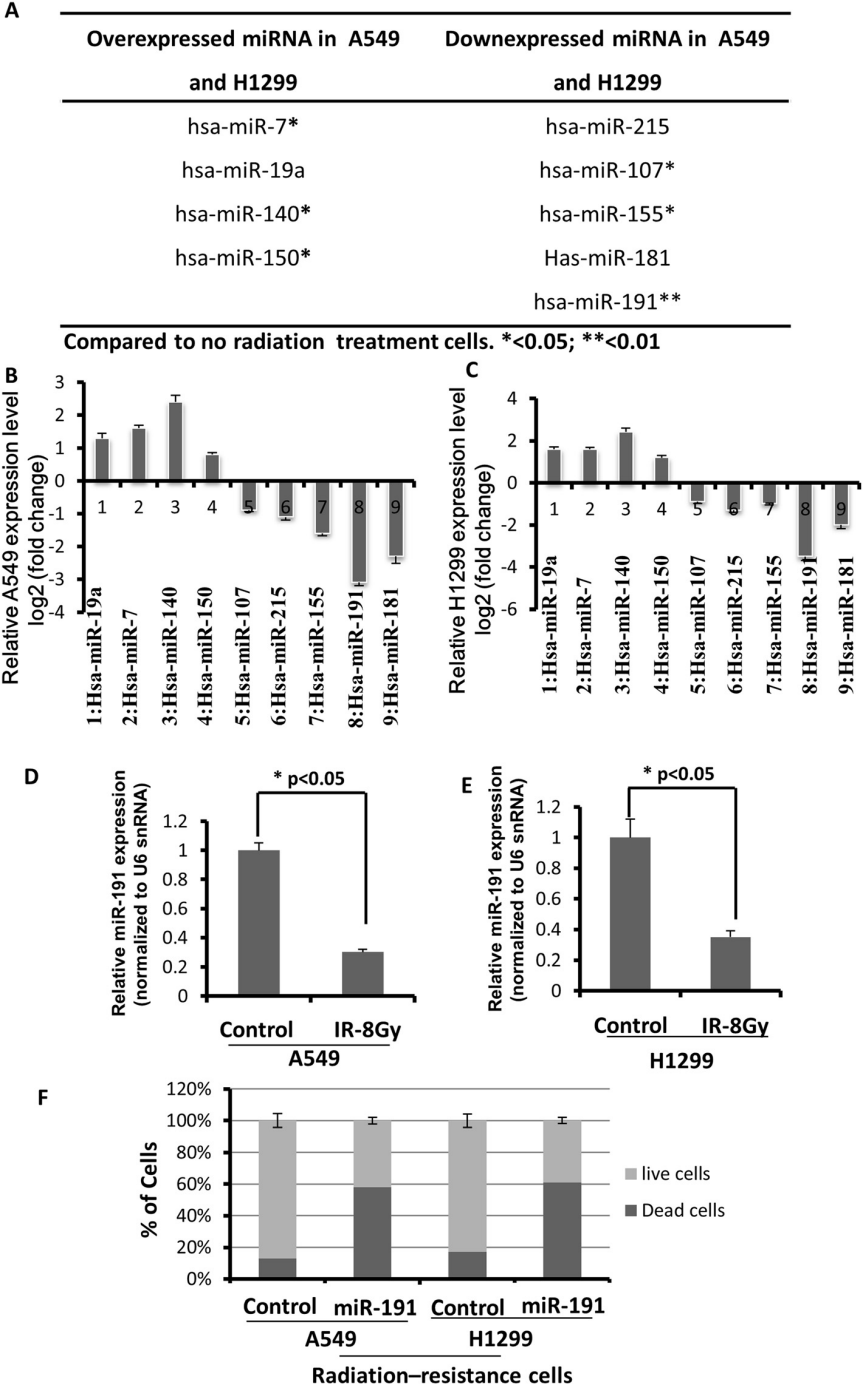
**miR-191 was functionally involved in the radiation resistance to A549 and H1299 cells. (A)** Micro-RNA profiles of radiation resistant and non radiation resistant A549 and H1299 cells were shown. Supervised hierarchical clustering of cell lines based on their differential miRNAs expression with ΔLMR > 2 between the two groups were exhibited. Each column represents a cell line and each row a probe set. **(B-C)** Relative miRNAs expression in A549 and H1299 cells in **(A). (D)** Downregulation of miR-191 expression in radiation resistant A549 compared to non-radiation resistant A549 cells. Results were normalized to snU6 expression level and represented as mean ± S.E. from three independent replicates. (*p <0.05 vs control, n = 3). **(E)** Downregulation of miR-191 expression in radiation resistant H1299 compared to non-radiation resistant H1299 cells. Results were normalized to snU6 expression level and represented as mean ± S.E. from three independent replicates. (*p <0.05 vs control, n = 3). **(F)** Cell viability after infected with Lenti-virus-miR-191 for 48 h.

Up and down-regulated miRNAs were listed based on high-throughput microarray assay in Figure 3A. Among all the miRNAs analyzed, miR-7, miR-140, miR-150, miR-107, miR-155, miR-191 were found to be significantly up or down-regulated in radiation-resistant compared to non-treatment cells (Figure 3B and C). Dysregulated miR-191 has been discovered in various types of human tumors such as breast, prostate [33] and colorectal cancer [34]. miR-191 is a highly conversed molecule and has been found dysregulated in more than 20 cancer types [35]. miR-191 is highly overexpressed in lung cancer patients and is suggested to be an oncogenic miRNA [33]. It is also an excellent candidate for disease diagnosis as it is non-invasive and widely spread in human serum or saliva [36]. Thus, miR-191 represented the most significant difference and therefore drew an attention for further analysis in our study. The comparisons of relative miR-191 levels by Taqman qRT-PCR between radiation-resistant and non-radiation treated cells were conducted and results are shown in Figure 3B and C. Results were normalized to snU6 expression level and represented as mean ± S.E. from three independent replicates. As shown in Figure 3B and C, there is significant reduction of miR-191 expression level in both radiation resistant A549 and H1299 cells. In order to whether miR-191 levels would influence cell viability, radiation-resistant cancer cells were infected with Lenti-virus-miR-191 to overexpress miR-191. Figure 3D shows that with miR-191 overexpression and radiation-resistant treatment (8 Gy) for 3 weeks in A549 and H1299 cells, viability of residual cells was decreased to 40% of the negative controls without miR-191 overexpression. These results indicated that miR-191 overexpression plays a key role in radiation-resistance in A549 and H1299 lung cancer cells.

### Association between miR-191 and autophagic marker

**Figure 4:**
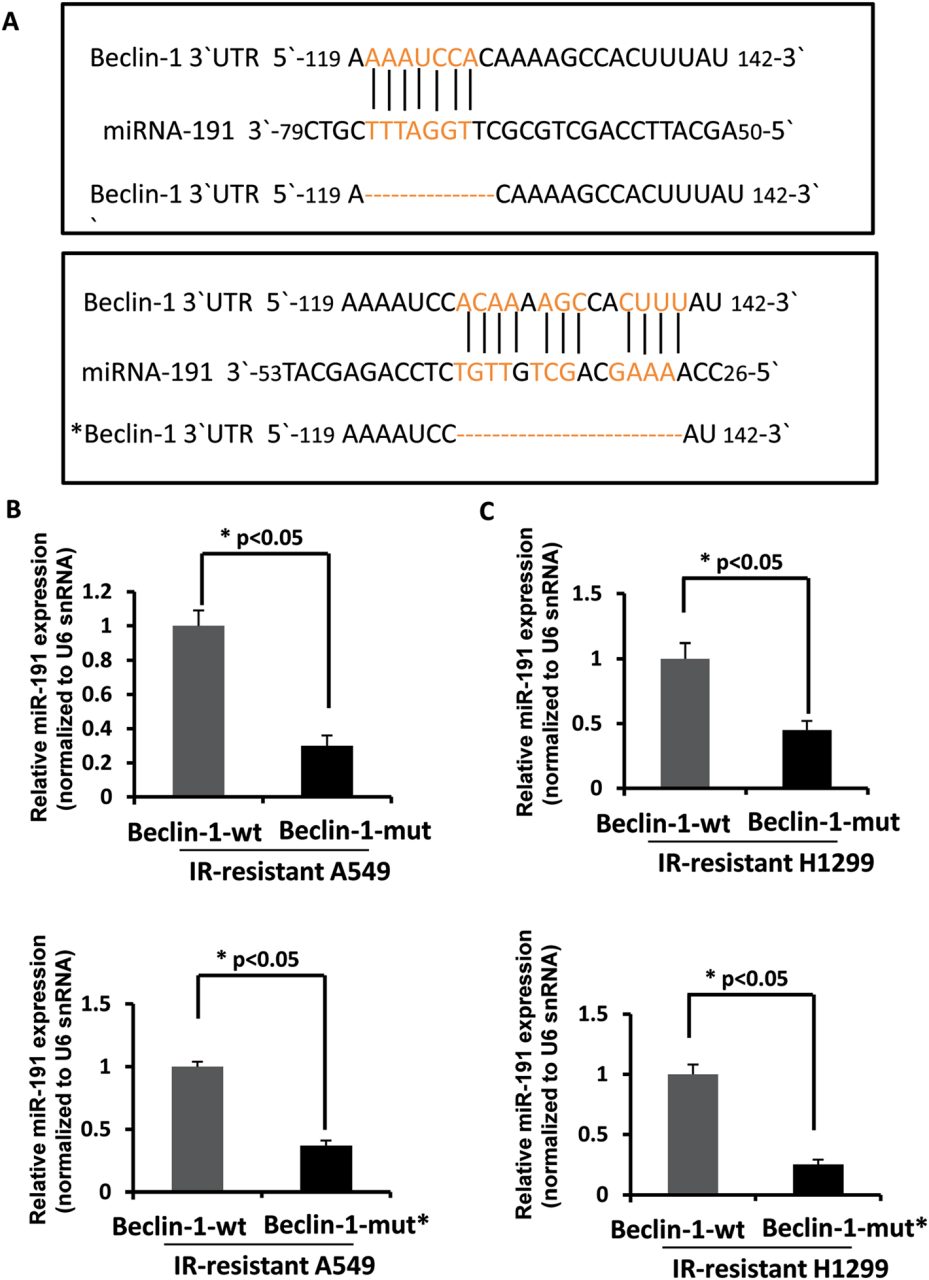
**Beclin-1 was a direct target of miR-191. (A)** Schematic representation of the 39- UTR of human Beclin-1 transcript. Predicted miR-191 binding site was depicted. The numbers (+121–140) represented the nucleotides that were predicted to base pair with the miR-191 seed sequence. **(B)** The expression of miR-191 in radiation-resistant A549 cells with or without Beclin-1 mutation (*p <0.05 vs control, n = 3). **(C)** The expression of miR-191 in radiation-resistant H1299 cells with or without Beclin-1 mutation (*p <0.05 vs control, n = 3).

We next investigated if there is any link between miR-191 and autophagic maker proteins. Potential binding sites between miR-191 seed sequence and Beclin-1 sequence were predicted by comparing base pairs of each. Two complementary binding sites were detected which indicate potential binding affinity between Beclin-1 and miR-191. Those two binding sites were then knocked out for further infection treatment. Radiation-resistant cells were infected with Lenti-virus-Beclin-1wt and mut (two mutation sites of Beclin-1) respectively (Figure 4A). Figure 4B and C showed there were relatively lower expression levels of miR-191 in Beclin-1 mut infected cells, suggesting decreased binding affinity between miR-191 and Beclin-1 as a result of removal of binding sites. Therefore our result indicated potential pathological association between miR-191 and autophagic marker protein Beclin-1 in lung cancer cells.

### MiR-191 modulated Beclin-1 expression

**Figure 5:**
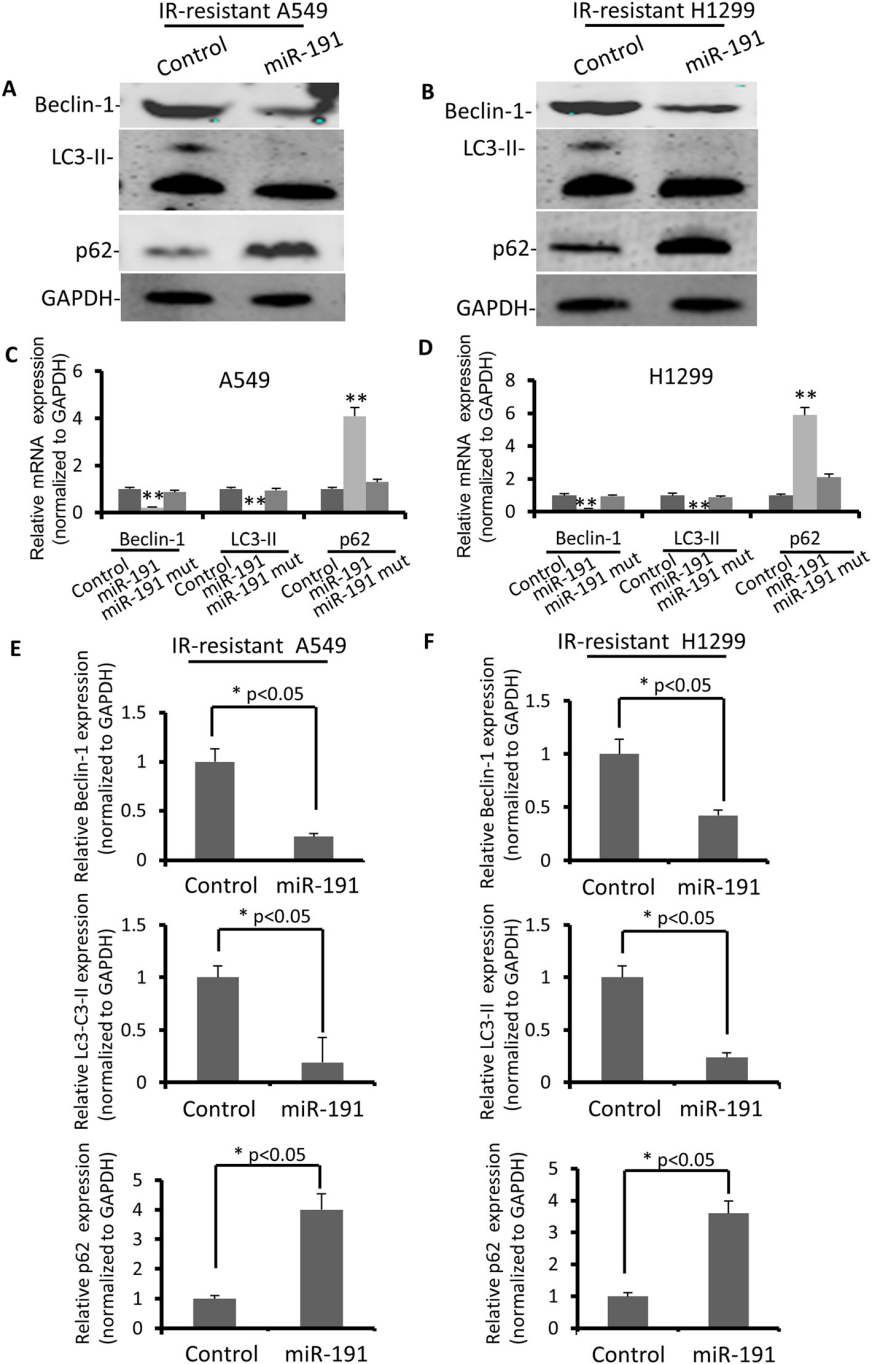
**miR-191 modulated Beclin-1 expression in radiation resistant A549 and H1299 cells. (A)** Expression status of certain autophagic marker proteins Beclin-1, LC3-II, p62 and GAPDH (loading control) in miR-191-overexpressed A549 cells. **(B)** Expression status of certain autophagic marker proteins Beclin-1, LC3-II, p62 and GAPDH (loading control) in miR-191-overexpressed H1299 cells. **(C)** Relative Beclin-1, LC3-II and p62 mRNA expression levels in miR-191 mutant A549 cells. bars represent mean ± S.E. (*p <0.05 vs control, n = 3). **(D)** Relative Beclin-1, LC3-II and p62 mRNA expression levels in miR-191 mutant H1299 cells. bars represent mean ± S.E. (*p <0.05 vs control, n = 3). **(E)** Relative Beclin-1, LC3-II and p62 mRNA expression levels in miR-191 overexpressed A549 cells, bars represent mean ± S.E. (*p <0.05 vs control, n = 3). **(F)** Relative Beclin-1, LC3-II and p62 mRNA expression levels were quantified by qRT-PCR analysis in miR-191 overexpressed H1299 cells, bars represent mean ± S.E. (*p <0.05 vs control, n = 3).

Recent study has revealed there are more radiation resistant cancer cells appearring along with hypoxia-mediated autophagy, suggesting the existence of autophagy-mediated stress resistant mechanism for cancer cells [37]. It is reasonable to speculate that inhibition of miR-191 expression in lung cancer cells would contribute to the establishment of radiation-resistant cells via mediating cellular autophagy. *Vice versa*, elevated miR-191 might also interfere with the forming of stress resistant cancer cells. The next step is to study whether autophagic activity would be affected if expression level of miR-191 increases in radiation-resistant lung cancer cells. The data demonstrated reduced amount of Beclin-1 and Lc3-II and increased p62 level in cells overexpressing miR-191 via western blot (Figure 5A and B) as well as qRT-PCR (Figure 5C and D), which suggested reduced autophagy level with overexpression of miR-191. These data further verified that miR-191 target autophagy associated Beclin-1 via two base pairing sites to interfere with autophagy. Therefore, miR-191 is a biomarker for cancer diagnosis, prognosis, and moreover serves as a potential target for the prevention of tumor recurrence after radiotherapy.

To summarize our study, we performed high-throughput microarray assay and found significantly decreased level of miR-191 in radiation-resistant cells than normal non-treatment cancer cells. Base-paired sequences between miR-191 and Beclin-1 reveal certain correlation between them. Taken together we speculate that miR-191 level reduces in survived radiation-resistant lung cancer cells, leading to increased autophagy level. Our study provides evidence supporting the role of miR-191 as potential therapeutic target in treating of lung cancer, especially in radiation-resistant cases in the future.

## Methods

### Cell culturing and proliferation

Cell culturing and established models of radiation-resistant cell lines A549 and H1299 cells were harvested by exposing to trypsin. Cell suspensions were irradiated using a ^137^Cs gamma-ray source with doses ranging from 0 to 12 Gy and plated in 6 well plates, 500 cells per well. Cultures were incubated at 37°C in 5% CO_2_. Optical density of each group with different dosages of radiation was detected at 7 times by using CCK-8 (Day 2, 4, 7, 10, 14, 18 and 21). The 8 Gy was chosen as a standard radiation dosage for the next test. Both normal and 8 Gy radiation-treated A549 and H1299 after 3 weeks were stained with DAPI (blue) for cell viability. Images were acquired under fluorescent microscopy.

### CCK-8 staining

CCK-8 staining was used to detect cell growth conditions. 100 μl of cell suspension (5000 cells/well) obtained from 2, 4, 7, 10, 14, 18 and 21 days after radiation treatment were dispensed into a 96-well plate. This plate was pre-incubated for 24 hours in a humidified incubator (e.g., at 37°C, 5% CO_2_). 10 μl of various concentrations of substances were added to the plate to be tested. The plate was then incubated for an appropriate length of time (e.g. 6, 12, 24 or 48 hours) in the incubator. 10 μl of CCK-8 solution was added to each well of the plate. Incubate the plate for 1–4 hours in the incubator. Measurement of the absorbance at 450 nm was performed by a microplate reader.

### Western blot

Expression levels of Beclin-1, LC3-II and p62 were determined by Western blot. Cells were harvested and lysed with lysis buffer (RIPA, Abcam). 20 μl 2 × sample loading buffer was added into each sample (0.125 M of 5 M Tris–HCl, amresco; 20% glycerol, usb; 4% of 10% sodium dodecyl sulfate, amresco; 1% β-mercaptoethanol, amresco; 0.2% of 0.05% (w/v) bromophenol blue, sigma). GAPDH was used as a loading control. 10% running gel (25% of 40% acrylamide stock, Beyotime; 0.375 M of 1.5 M Tris–HCl, pH 8.8; 1% of 10% sodium dodecyl sulfate; 1% of 10% ammonium persulfate; 0.1% tetramethylethylenediamine) was utilized for samples that have been boiled for 5 minutes. The gel was transferred to a same size membrane (Nitrocellulose transfer membrane, Protian) within transfer buffer (25 mM Tris base, 192 mM glycine, 0.037% sodium dodecyl sulfate, and 20% methanol) under 45 V for 40 min. The membrane was then incubated in primary antibody (Beclin-1, GAPDH, Abcam) with a 1/1000 dilution in blocking buffer (50 mM Tris base; 100 mM NaCl; 0.02% Tween 20; and 3% BSA) overnight. The membrane was rinsed by TTBS (0.1% Tween 20, 10 mM Tris base, 100 mM NaCl, pH 7.5) for three times before adding secondary antibody (Abcam) with 1/5000 dilution in blocking buffer for 2 hours. Background noise was reduced by careful wash. The results were visualized using ECL kit (Abcam) and observed by GeneGnome mechine (Syngene).

### Real-time RT-PCR

mRNA of Beclin-1, LC3-II and p62 from cells were extracted by RNeasy Mini Kit (Qiagen). NanoDrop 8000 spectrophotometer (Thermo Scientific) was used to determine the quality and quantity of extracted RNA. 1000 ng RNA was used for each reaction to produce cDNA using high capacity cDNA reverse transcription kit (Applied biosystems) following manufacturer’s instructions. The reaction was initiated at 25°C for 5 min followed by annealing at 50°C and subsequent elongation at 70°C. cDNA products were then diluted by adding DNase and RNase free water to 250 μl and frozen at −20°C for further gene expression assay. 10 μl 1 × PCR master mix, 5 μl diluted cDNA, 4 μl water and 1 μl probe were mixed together in every reaction. Gene expression was measured with quantitative real-time RT-PCR system. Taqman real-time RT-PCR was performed to specifically detect the relative levels of miR-191 in radiation resistant and no radiation resistant H1299 for higher sensitivity.

### Micro-RNA profiles in radiation-resistant cells

Overexpressed and downexpressed miRNAs in both A549 and H1299 were screened and selected based on miRNA microarray assay. Taqman real-time qRT-PCR was performed to detect the relative levels of miR-191 in radiation resistant and non-treatment cells. miR-191 was normalized against U6 spliceosomal RNA (U6 snRNA).

### Cell viability evaluation with additional miR-191

Radiation resistant A549 and H1299 cells were infected with Lenti-virus-miR-191 for 48 h. The cells with or without treatment of Lenti-virus-miR-191 were subjected to FACS analysis after being stained by Trypan blue for the measurement of cell viability (n = 3).
